# Contamination Levels and Identification of Bacteria in Milk Sampled from Three Regions of Tanzania: Evidence from Literature and Laboratory Analyses

**DOI:** 10.1155/2017/9096149

**Published:** 2017-08-08

**Authors:** G. Msalya

**Affiliations:** Department of Animal, Aquaculture and Range Sciences (DAARS), Sokoine University of Agriculture (SUA), P.O. Box 3004, Morogoro, Tanzania

## Abstract

Milk in Tanzania has been reported to be contaminated with large number of bacteria. This is because (1) milk is obtained from animals with unknown health status, (2) good milking and handling practices are to a large extent not observed, and (3) marketing and distribution are done in informal channels. These factors are potential causes of milk-borne diseases and milk quality loss. The aim of this study was to assess nutritional risks in milk as reported in literature over a period of 20 years and through analyses of samples collected during the present study. The issues highlighted in literature were high bacteria and coliform counts exceeding standard levels in East Africa, prevalence of bacteria and drug residues in milk, and adulteration. Based on performed analyses, total bacterial count 1.0 × 10^7^ colony forming units per millilitre (cfu/ml) and total coliform count 1.1 × 10^7^ cfu/ml, also greater than recommended levels, were found. Ten bacteria types were isolated from milk samples (five,* Pseudomonas aeruginosa*,* Listeria monocytogenes*,* Listeria innocua*,* Listeria ivanovii*, and* Klebsiella *spp. are reported in Tanzanian for the first time). Two drugs tetracycline and sulphur were detected. Therefore, it is worth noting that integrated research is needed to evaluate the situation and address these challenges.

## 1. Introduction

Milk is of great importance particularly in the rural communities of Africa as a source of macro- and macronutrients that improve the nutritional status of individuals and populations. Also, it is one of the pathways out of poverty for millions of people in these communities [1]. In Africa consumption of milk and milk products will continue to increase from their current levels as a result of population increase, economic growth, and urbanisation [2]. A larger contribution to the gross domestic product (GDP) has been predicted [3]. In Tanzania milk production increased by 130% over the last decade to about 1.8 billion litres per year in 2011 and the contribution to the agricultural GDP was significant [4]. Until 2015, the per capita consumption was approximately 45 litres per annum and milk is nick named “white gold” because of importance [5]. However, in Tanzania a large percent of milk (70 percent) is obtained from the indigenous cattle, namely, Tanzania shorthorn zebu (TSZ), which are distributed throughout the country and comprise about 95 percent of all 25 million cattle. These animals are neither inspected nor vaccinated against diseases and sometimes they are treated locally with their owners and withdrawal periods are not observed. The remaining percent is obtained from the crossbred animals (dairy breeds × TSZ) which are less than one million and are kept by small-holder farmers in urban and periurban areas in a semi-intensive system with improved management including vaccinations, even though inspection of the animals is also rare [5]. A very small amount of milk is obtained from goats and sheep which are also found in the traditional or small-holder farming systems with very low inputs. In addition, milk and milk products are mainly sold in informal market channels including open markets, farm gate or neighbours, door-to-door sales, and small shops operated by vendors or hawkers where producers and consumers meet. In many rural households, milk and milk products are produced for consumption at home farms. At least 80% of milk consumed off-farm stems directly from farmers to consumers [6]. In these channels, there is less regulation, rarely inspected traditional products predominate, actors are not licensed, and, in some occasions, products from sick animals may be consumed [7]. These circumstances are linked to contamination of milk with high levels of bacteria, cause loss of nutritional quality of milk, and are probably the greatest challenges of the dairy industry in the country.

Government of the United Republic of Tanzania has made several attempts to improve safety and standards of milk as well as the performance of markets. According to the Tanzania Dairy Board (TDB), regulation of the dairy industry in Tanzania started during the colonial era in 1920s (http://www.tdb.go.tz). Between 1970 and 1980s, collection, processing, and marketing of milk were the responsibility of one major parastatal organization, known as Tanzania Dairies Limited (TDL). However, performance of the seven processing plants under TDL was low leading to either their closure or privatization. At present, the private commercial processing plants including Tanga Fresh in Northern Tanzania, Tan-Dairies in the business capital of Dar es Salaam, ASAS Dairies and CEFA in the Southern highlands, and International Dairy Products in Arusha region operate at 20 to 75% of their plants capacity thereby processing only 31% of the milk produced in the country and the only amount which may be inspected [5]. In 2003, a Food Law was revised and placed food safety control activities under the Tanzania Food and Drugs Authority [16]. However TFDA does not use any documented formal risk assessment methods, for example, those suggested by the Codex Alimentarius Commission (CAC) or the Organization of Animal Health (OIE). In 2005, TDB was established and given a mandate of developing, regulating, and promoting dairy industry in Tanzania. Moreover, the traceability system ratified by the government and stipulated in national legislation in 2010 is still under development. As such, contamination of milk and related nutritional risks are increasingly reported in literature.

In the last five years a greater number of literatures reported bacterial count, for example, between 3 × 10^6^ colony units per millilitre (cfu/ml) in [11] and 5.4 × 10^6^ cfu/ml in [14]. According to [17], these values exceed the levels acceptable in the East African community (EAC) countries. This condition causes increased number of bacteria in the milk and contributes to loss of milk quality. Between 2006 and 2014, a greater number of pathogens including* Brucella (B*.*) abortus*,* Campylobacter *spp.,* Clostridium *spp.*, Escherichia (E*.*) coli*,* Corynebacterium *spp.,* Leptospira *spp.,* Mycobacterium *spp.,* Salmonella *spp.*, Staphylococcus (S.) aureus*, and* Streptococcus (S.) agalactiae* were isolated from milk [10, 14, 18, 19] and were linked to incidence of diseases such as tuberculosis (TB), brucellosis, diarrhoea, typhoid, rift valley fever, and allergies reported among milk consumers in different places in the country [14, 20]. The prevalence of these pathogens in the milk was associated with the occurrence of diseases in the animals including cow mastitis [21, 22], goat mastitis [23], bovine TB (BTB) [8], and brucellosis [19]. At the same time, a number of drug residues were reported along the milk value chain at least in some locations of Tanzania [6, 15]. The effects of drug residues may include tolerance or resistance of bacteria to antibiotics [9, 18] and antibiotic resistant consumers due to consumption of contaminated milk [24]. They may also cause negative health effects such as allergies in the consumers [25, 26]. Furthermore adulteration has also been listed among the major challenges and was shown to affect nutritional and processing quality (loss of quality) and increase chemical or microbial contamination. Specifically, [11, 15] showed that high adulteration reduced specific gravity of milk in Tanzania and introduced microbial hazards into milk as also suggested by [27]. In summary, these conditions lead to low nutritional quality of milk and cause a potential burden of milk-borne diseases thus negatively affecting consumers. Therefore this study was carried out to assess the trend by reviewing the nutritional risks reported in literature and analyzing new milk samples to study the current situation and to summarize both (literature and laboratory results) for interpretation and suggestion of research opportunities for reducing the risks.

## 2. Materials and Methods

### 2.1. Systematic Search of Literature (SSL)

The first part of this study was the systematic search of literature (SSL) to assess and summarize the effects of milk contamination reported in the country over the last 20 years. The SSL involved identifying a need for the search (status of milk bacteriological quality in Tanzania), selecting the literature databases for searching (key engines for search decided), designing the search and literature inclusion criteria, and screening and revision, as well as data extraction and reporting. Detailed steps for scientific SSL were suggested by [28]. In this study seven key words (antimicrobials, brucellosis, mastitis, milk contamination, milk pathogens, TB, and Tanzania) were used for searching literature relevant to subject in five search engines, namely, google, PubMed, Web of Science/Web of Knowledge, CAB Direct, and African Journal Online (AJOL). Only papers from peer reviewed sources (journal, conference proceedings, theses, or book chapters) were downloaded and reviewed. From the papers, information was extracted, interpreted, and summarized as results.

### 2.2. Milk Sampling

The second part involved sampling milk for laboratory analyses on selected aspects of quality or contamination, including identification of target pathogens and testing of drug residues. A total of 328 raw (fresh) milk samples from selected places in three regions of Tanzania (Morogoro, Coast, and Tanga) were collected for this study. Approximately 50 mls of for each sample was aseptically obtained from bulking containers in selected farming households during morning milking using sterilized falcon tubes. These samples were cooled on a field ice packed cool boxes and were sent to the refrigerator after sampling (about 4 to 6 hours). The collection was done by SUA undergraduate (B.S.) students or technicians trained by a project, namely, Safe Food Fair Food (SFFF) II, which was implemented in Tanzania by researchers from the International Livestock Research Institute (ILRI) based in Nairobi, Kenya, and the Sokoine University of Agriculture (SUA) based in Morogoro, Tanzania. The sampling sites were either a selection of the SFFF II project (Morogoro and Tanga) according to the project criteria [7] or request of farmers associations in one region (Coast).

### 2.3. Laboratory Analyses

Four types of analyses were carried out at SUA. First, within 48 hours samples were analyzed for total bacterial count (TBC) and total coliform count (TCC) using conventional laboratory methods. Secondly, pathogens were identified by laboratory procedures according to the relevant protocols of the International Standard Organization (ISO) or DNA genotyping for one pathogen. Initial isolation procedures were performed according to ISO 4833-1 protocols [29] and were followed by detection and confirmation of target microorganisms based on colonial morphologies as well as confirmatory tests using specific commercial kits. For example, identification of* Salmonella *spp. was performed according to ISO 6579 protocol [30] and confirmation was accomplished using* Salmonella* Test kit (Oxoid® Ltd., Basingstoke, Hampshire, England) according the instructions of the manufacturer. The procedures in ISO 11290-1 [31] were followed during identification of* Listeria (L.) monocytogenes *and confirmation tests were done using Listeria Test kit (Oxoid Ltd., Basingstoke, Hampshire, England) according to instructions of the manufacturer. The* S. aureus* and* E. coli* were identified using the procedures in ISO 6888-1 [32] and ISO 21528-2 [33], respectively. More specific tests for others pathogens were performed and confirmed with relevant ISO protocols and confirmatory kits. For some pathogens, in particular*, Pseudomonas (P.) aeruginosa *and* Proteus *spp. different sugars were used as biochemical tests.

Thirdly, polymerase chain reaction (PCR) option was used to directly detect* B. abortus* in milk. This method was preferred to conventional or ISO procedures because of its reliability and specificity in detecting target species particularly in a mixture of microorganisms associated with cattle [34, 35]. The PCR was performed using DNA samples from* B. abortus* (positive control obtained from test samples elsewhere) provided by the Genome Centre of SUA and DNA purified from test (milk) samples. Milk DNA was purified from 1 ml of raw milk according to the standard phenol-chloroform also with isoamyl alcohol at 24 : 24 : 2 with 0.5 M guanidinium thiocyanate as detailed in [36]. To avoid contamination and to achieve purity of the DNA as earlier suggested [36], procedures for breaking down fats and proteins using lipase-phospholipase solution and trypsin solution, respectively, several washing steps in double distilled water (ddH_2_O), and incubations at relevant temperatures were performed. Also, a second extraction (reextraction) from first DNA after suspension in sodium dodecyl sulfate (SDS) was done using chloroform-isoamyl (24 : 2) followed by cleaning in 100 percent alcohol, drying, and reconstitution in 50 *μ*l of ddH_2_O.

A pair of primers 5′TCGAGAATTGGAAAGAGGTC 3′ and 5′ GCATAATGCGGCTTTAAGA 3′ [36] sequences of the 726 bp long 16S–23S rRNA gene of* B. abortus* in positive and test DNA samples were used in the PCR. The 25 *µ*l reaction mixture included 12.5 *μ*l of reaction buffer, 8 *μ*l of RNase free water, 10 pmol for each of forward and reserve primers, 0.5 *μ*l of Taq DNA polymerase (Invitrogen Carlsbad, CA), and 2 *μ*l DNA. The reaction mixture with 2 *μ*l ddH_2_O in place of DNA was used as a negative control. The amplification condition was 30 cycles of denaturation at 95°C for 15 seconds, annealing at 50°C for 30 seconds, and extension at 72°C (60 seconds) run by a StepOne PCR (Applied Biosystems). PCR products were separated on 1.5% agarose gels and visualized on a parafilm while being loaded with 1 *μ*l loading dye (Promega, Madison, USA) and ethidium bromide staining under ultraviolet light. Detection was based on occurrence of bands at the target location of 16S–23S rRNA gene.

Fourthly, the presence of drug residues was determined with the Charm EZ antimicrobial inhibition assay screening kit (Charm Sciences, Inc., Lawrence, Mass) according to the manufacturer's instructions and as described in [6]. In the present study, only two antimicrobials (sulphonamide and tetracycline) were determined. These are the major drugs commonly used by farmers in Tanzania. Detection was performed after incubation for 8 minutes for tetracycline and 4 minutes for sulphonamides using a negative control, that is, milk sample obtained from animals believed not to have received any treatment with antibiotics one month before the time of analyses. The positive controls were tetracycline and sulphonamide tablets present in the commercial kit which were used after dissolving in 1 ml of the antibiotic-free milk. Thereafter 30 *µ*ls (acceptable maximum) of each sample was stripped into the Charm EZ machine and readings were recorded. The samples were confirmed as contaminated with drugs using a score (1–5) whereby 4 and 5 were the positive numbers (scores).

### 2.4. Data Analysis

Data from literature were summarized into identified variables in excel and summarized into information of interest in the present study. List of literatures contributing to the extracted data is also provided. From present analyses, the TBC and TCC were averaged per region and are presented as cfu/ml. To report on contamination with specific bacteria type, the number of samples positive for identified pathogen was computed as percentage in the total positive samples per region. Totals for all regions were also obtained. The prevalence of* B. abortus* was analyzed as percentage of positive DNA samples (those which showed bands for a target segment or sequences of 16S–23S rRNA gene of* B. abortus*) on PCR. The number of samples detected with antimicrobial residues was counted manually and summarized as percentage of all positive samples per region. Statistical analyses involved comparisons of averages or totals of TBC, TCC, number of positive samples for identified bacteria, and positive antimicrobial residue samples (the dependent variable) against the sampling sites in this case the three regions (the fixed variables) using the General Linear Model (GLM) procedure of the Statistical Analysis System (SAS), SAS® Proprietary Software, Release 8.2 (SAS Institute Inc., Cary, NC, USA).

## 3. Results

### 3.1. Status of Milk in Tanzania for Last 20 Years: Evidenced from Literature

In total 45 articles reporting on the nutritional risks of milk and contamination of products in the dairy value chain in Tanzania since the last 20 years were reviewed. However, data presented here are summarized from only ten publications which involved milk as samples in the evaluations. The rest of articles were either surveys of the value chain (interviews of stakeholders or observation of the situations) or detected pathogens in other biological samples, for example, blood samples or other tissues and therefore results from these articles were not extracted. Four major nutritional issues and risks in milk are extracted from literature, and these are (1) high levels of bacterial count to a greater extent exceeding levels acceptable at least in EAC countries, (2) large number of pathogens isolated in milk samples including samples obtained in markets or milk aimed for consumption, (3) antimicrobial (drug) residues that have been detected in the milk, and (4) adulteration that has been detected and was shown to lower nutritional quality of milk and cause further infection with microorganisms (Table 1). It is shown that, over the last twenty years, TBC values ranging from 8.4 × 10^4^ to 4.8 × 10^7^ cfu/ml were reported. At the same time, TCC values ranging from 1.4 × 10^6^ to 4.2 × 10^6^ cfu/ml were reported. Also, the bacteria* Mycobacterium (M.) tuberculosis*, atypical* Mycobacteria*,* Mycobacterium *spp.,* S. agalactiae*,* Arcanobacterium (A.) pyogenes*,* Staphylococcus (S.) epidermidis*,* Staphylococcus (S.) hyicus*,* Staphylococcus (S.) intermedius*,* Staphylococcus (S.) saprophyticus*,* S. aureus*,* Micrococcus *spp.,* Mucor *spp.,* B. abortus*, and* Corynebacterium *spp., as well as other organisms such as* Aspergillus *spp.,* Pseudomonas *spp., and yeast, were isolated from milk. Furthermore, some of the reported (detected) antimicrobial residues were shown to be above the recommended maximum residual limits, that is, higher than the minimum acceptable values (Table 1). These studies were conducted in seven administrative regions (about one-third) of Tanzania listed in Table 1.

### 3.2. Bacteria Count in the Present Study

To reassess the situation at the moment, a total of 327 milk samples were evaluated on the levels of TBC, TCC, and percentage contamination of two commonly used drugs (tetracycline and sulphur). Greater ranges in TBC and TCC were observed among regions from the lowest 0.06 × 10^7^ in Coast region samples to the greatest 2.30 × 10^7^ in Morogoro region samples. Regarding the TCC a similar situation can be noticed although the lowest TCC level was recorded in Tanga region samples. Concerning drug residues, more samples were positive in Morogoro compared to Coast region for both tetracycline and sulphur. There was no detection of these in Tanga samples. Overall results for TBC and TCC as well as total number of samples detected with drugs and percentage contamination are shown in Table 2. The differences in TBC and TCC levels as well as the number of samples positive for two drug residues among regions were statistically significant (*P* < 0.05) in regions.

### 3.3. Bacterial Isolation

**Figure 1:**
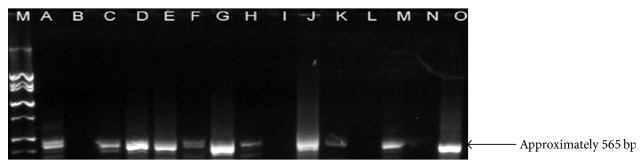
Detection of* B. abortus* in milk samples (16S–23S rRNA gene in* B. abortus*). M: ladder marker; B, I, and L: negative samples; A, C-H, J, K, and M: positive samples; D, E, and N: negative control; O: positive control.

In this study, the total number of positive samples was 238 with more positive samples in Tanga (87/103 or 70%) compared to other regions. In positive samples, ten groups or species of bacteria were identified. These were* E. Coli*,* Salmonella *spp.,* Klebsiella *spp., and* Proteus* spp. (four groups of bacteria not identified to species level) in addition to six species including* P*.* aeruginosa*,* L. monocytogenes*,* L. innocua*,* L. ivanovii*,* S. aureus*, and* B. abortus*. The* L. monocytogenes* was obtained in a greater number of samples than any other bacteria in all regions. Some bacteria were not detected in some regions. Number of positive samples per region and specific for each pathogen (bacteria) and their percentages in the positive samples are presented in Table 3. Moreover, 57 of 238 equal to 23.9% positive milk samples (Table 3) were linked to contamination with* B. abortus* following the amplification of close to 600 bp (Figure 1) segment (sequences) of the 16S–23S rRNA gene of the species. Tanga region has a high prevalence of these sequences compared to Morogoro and Coast regions.

## 4. Discussion

The truth that contamination of milk particularly in the traditional farming systems and in the informal milk markets is high was reconfirmed in this paper using information from literature and additional analyses. Concerns of consumers being exposed to different forms of milk hazards and associated risks have been reported regularly in the last two decades [6, 8]. Evidences gathered in this study indicate that the levels of milk contamination and nutritional risks are on the rise. It can be shown that the levels of bacterial and coliform count reported in previous reports, for example, TBC values of 5.4 × 10^6^ cfu/ml in [14], 3.3 × 10^5^ cfu/ml in [15], and TCC between 1.4 × 10^6^ and 4.2 × 10^6^ cfu/ml, as shown by [11] are higher compared to the levels accepted at least in EAC countries. In EAC, the recommended levels are 2.0 × 10^5^ and 5.0 × 10^4^ cfu/ml for TBC and TCC, respectively [17]. Analyses performed in this study also revealed high levels of TBC and TCC. In this regard, greater percentage of Tanzanian milk is microbiologically of very poor quality.

At the same time, the number of isolated bacteria is on the rise and cases related to milk contamination have increased in recent years. For example, one study [20] showed that 22.9% of diarrhoea cases in hospitalised children in business capital of Tanzania (Dar es Salaam) were due to milk* E. coli*. In another study,* M. bovis* was confirmed to be positive in humans diagnosed with TB and these came from households owning cattle infected with BTB [37]. Ten bacteria types were isolated in milk during analyses performed in this study. To the best knowledge of the author, five bacteria (*P. aeruginosa*, three* Listeria *spp.* (L*.* monocytogenes*,* L*.* innocua*, and* L*.* ivanovii),* and* Klebsiella *spp.) have not been reported and are therefore reported in milk for the first time in dairy value chain in Tanzania. However* P. aeruginosa* was previously reported in isolates from patients in Muhimbili National Hospital, in Dar es Salaam [38] while* Klebsiella *spp. were recently detected from rectal swabs in other domestic animal species (including dogs and pigs) in the Lake Zone of Tanzania [39]. These and other bacteria have also been reported in the informal milk markets elsewhere in EAC [40] and have been shown to elevate the nutritional risks of milk. For example* Listeria *spp. may both affect humans (causing ill-health conditions) and poison the feeds.* L. monocytogenes* may cause death in immunologically deficient persons [41] while* L*.* ivanovii* is associated with abortions in ruminants [42] and bacteremia in immunodeficiency debilitated patients [43].* S. aureus* produces enterotoxins and has many effects in human including, for example, infertility in males and females [44, 45], has been associated with mastitis in animals [9], and is probably the most reported bacterium in dairy value chain of Tanzania.* E.coli* species are reported in high numbers in many countries and represent a threat to food safety [46] and are mainly contributed by fecal contamination from ruminants.

These and other risks in milk are contributed by many factors including, for example, animal diseases such as BTB, brucellosis, anthrax, mastitis, salmonellosis, and campylobacteriosis from which pathogens are shed in the milk [19, 21, 22, 47, 48]. It has also been shown that milk can be contaminated by unclean containers during milking, storage, collection, processing, delivery, or serving of milk as well as environmental factors such as unclean animal houses and contaminated feeds or water [49]. Occurrence of some of the pathogens such as* B. abortus* is not surprising and may increase with increased sample size because of the predominance of tradition cattle keeping and rearing under 100% extensive communal grazing fields also characterised by trekking with animals to different places, lack of a controlled breeding practice, disease knowledge and traditional treatment among pastoralists, and the way of marketing milk. Direct detection of* B. abortus* using PCR methods has been shown to be quicker and more accurate and can test for any* Brucella species* because they are genetically very similar [36]. Furthermore, antimicrobial residues were observed in milk samples in the present study. These render antimicrobial resistant pathogens which are finally shed in the milk, thereby resulting in a direct impact of antimicrobial resistance in consumers [6, 15]. This situation is associated with improper administration (use) of antibiotics by either farmers themselves or veterinary officers during treatment of animals. Moreover, adulteration has been shown to contribute to poor nutritional quality of the milk, for example, the reduction of specific gravity of milk as described by [11, 15].

**Figure 2:**
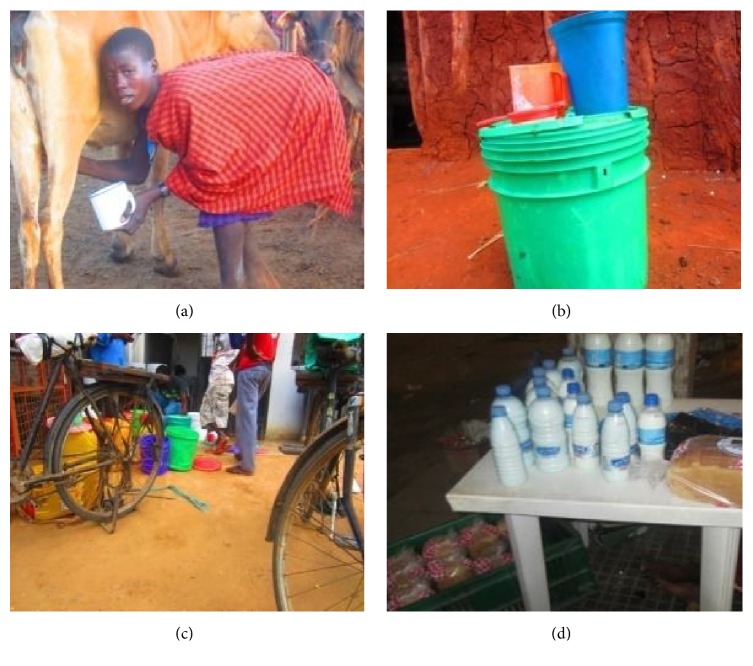
Pictorial presentation of a short milk supply chain in the informal dairy sector of Tanzania. (a) Hand milking. (b) Bulking or collection in plastic buckets. (c) Transportation by bicycle. (d) Selling in used water bottles.

In Africa food poisoning or at least contamination is increasing and causes great risks in consumers. Reference [50] estimated that food (including milk) and waterborne diarrhoeal illnesses contributed to 2.2 million of annual deaths in the continent of which 1.9 million were children (40% below five years). Greater levels of bacteria and coliform count such as those reported here have been reported in different places in Tanzania including in samples from the present study regions [14, 51, 52] and in other African countries [53–55]. In Tanzania probably the situation will worsen as more laboratory reports are expected from SFFF II (Brown et al., unpublished; Hyera et al., unpublished). Although consumers appreciate good quality and are aware of the risks, their choices especially among the poor socioeconomic groups are restricted and sometimes people cannot afford to discard milk even if it is of low quality because of poverty or gender related factors [56]. In one region of Tanzania, 83.8% of samples from marketed or market ready milk were detected with aflatoxins M1 (AFM1) at levels above 2.007 ng/ml exceeding the levels 0.05 ng/ml and 0.5 ng/ml set by TFDA and CAC, respectively [57]. It is therefore of great importance to find ways in which the risks can be reduced or alleviated by investing in short term plans such as training of key actors and establishment of a user friendly system of monitoring along the dairy value chain. It is possible to reduce the number of bacteria or coliform count to some levels down as was with the TBC and TCC levels in Tanga and Coast regions compared to Morogoro region in the present study. This can be associated with training programmes conducted by Tanga Dairy Cooperative Union (TDCU), the owner of Tanga Fresh Company in which quality is a major focus and all value chain stakeholders are involved in the training programmes. Moreover, in Tanga stern milk inspection is conducted and probably for these reasons, no antimicrobial residues were detected in Tanga milk samples. Also Coast region is the main source of milk for the processing plants and small processors in Dar es Salaam where also inspection has improved in recent years. In Morogoro region and overall in many places, to a large extent milk is still marketed in the informal markets and no much inspection is followed and therefore the whole chain is unsatisfactory (Figure 2). Another option would be such as investing in awareness creation for stakeholders, for example, to observe good handling practices and improvement on collection, storage, and marketing. Reference [6] suggested identification of incentives that could promote behavioural change in particular among farmers. Moreover, low rates of bacterial count, lower number of positive samples or identified pathogens, or nondetection (ND) in some region is a recommended step and efforts should be made to improve the situation. The short term solution for consumers at this moment is continuation of boiling milk before consumption and avoidance of taking raw milk traditionally processed products such as fermented milk, ghee, and local yoghurt.

## 5. Conclusions

This study has shown that contamination of milk with bacteria remains high since the last 20 years. The overall TBC and TCC values in this study were 1.0 × 10^7^ and 1.1 × 10^6^ cfu/ml, respectively, and exceed the standard levels for milk (2.0 × 10^5^ and 5.0 × 10^4^ cfu/ml, resp.) in EAC countries. At the same time a large number of bacteria were isolated from milk samples and have been associated with milk-borne illness (diseases) in the consumers. From analyses performed in this study, 238 out of 327 samples or 72.8 percent were infected with 10 different types of bacteria. Among these, five* P. aeruginosa*,* L*.* monocytogenes*,* L*.* innocua*,* L*.* ivanovii*, and* Klebsiella *spp. are reported in milk samples for the first time. In this regard Tanzanian milk is of poor microbiological quality. In addition, residues of two drugs (tetracycline and sulphur) were detected in 108 samples or 33 percent, implying that either milk producers do not observe the withdrawal periods after administering drugs or they add these in the milk to protect them from infections with microorganisms. However, drugs residues can have double effects by rendering the microorganisms tolerant if not used properly thereby increasing contamination of milk or they can also make consumers drugs tolerant. Moreover, adulteration is reported in the reviewed literature and has been linked to reduced nutritional quality of milk and increased microbiological or chemical contaminations. These conditions will continue to threaten the quality of milk in Tanzania and may have several negative effects on the consumers. Therefore, more integrated research is needed countrywide or in more zones to evaluate the situation and address these challenges. In particular, future studies should consider researching on causes of risks and contamination levels at each level of the value chain.

## Figures and Tables

**Table 1:** Nutritional risks identified in milk sampled in different places of Tanzania and reported in literature.

Zone or region	Form of milk samples	*N*	Risk and levels (TBC or TCC in cfu/ml)	Risks in consumers	Reference
Southern highlands (Iringa and Mbeya regions)	Fresh udder milk	805	*M*.* tuberculosis *(*n* = 2, 6.5%) Atypical *Mycobacteria *and *Mycobacterium *spp.(*n* = 29, 93.5%)	(1) TB (2) Atypical *Mycobacteria *and *Mycobacterium *potentially pathogenic in low immunity people	[8]

East (Coast and Morogoro)	Fresh udder milk	919	*S. agalactiae* (1.2%), *A. pyogenes *(0.2%), *S. epidermidis *(2.8%), *S. hyicus* (0.1%), *S. intermedius *(1.1%), *S. saprophyticus* (0.3%), *S. aureus* (1.7%), *Micrococcus *spp. (0.7%), *Mucor *spp. (2.4%), *Aspergillus *spp. (0.1%), Yeast (18.5%)	(1) Pathogen shed in milk due to udder infection (2) Potential fungal infection	[9]

Dar es Salaam and Mwanza	Fresh from containers	982	Antimicrobial residues (*n* = 353, 36%)	Exposure to unacceptable concentrations of antimicrobial residues (above maximum residue limits)	[6]

Dar es Salaam	Fresh from containers	128	TBC 8.2 × 10^6^ *E. coli *(6.3%), *Bacillus cereus *(6.3%), *S. aureus* (6.3%), *S. agalactiae *(6.3%), *Enterobacter aerogenes *(5.6%), *Enterococcus faecalis *(4.7%)	(1) High bacterial count (2) Pathogen shed in milk	[10]

Northern Coast (Tanga region)	Fresh from containers	59	TCC (up to 4.2 × 10^6^) *B. abortus* (*n* = 33, 56%) Adulteration	(1) Quality loss (2) Brucellosis (3) Quality loss	[11]

Eastern Coast (Dar es Salaam)	Fresh milk from containers	120	TBC (up to 4.8 × 10^7^) *E. coli* (83%) *S. aureus* (27.3%)	(1) High bacteria (2) Lowered quality (3) Contamination	[12]

Eastern Coast (Dar es Salaam)	Boiled milk from containers	22	TBC (up to 3.0 × 10^4^) *E. coli* (36.4%) *S. aureus* (22.7%)	(1) Boiled with reduced bacteria but still pathogenic	[12]

Northern Coast (Arusha region)	Fresh milk from sellers	75	*Salmonella *(*n* = 28, 37%)	Gastroenteritis	[13]

Northern Coast (Arusha region)	Fresh milk from containers	180	TBC (up to 1.5 × 10^7^) *S. aureus* (*n* = 31, 33%) *Corynebacterium*spp. (*n* = 10, 11%) *Pseudomonas *spp. (*n* = 10, 10%)	(1) High bacteria, lowered nutritional quality (2) Food poisoning	[14]

Eastern Coast (Morogoro region)	Fresh and boiled milk from containers	201	TBC (up to 3.3 × 10^5^) Adulteration Antimicrobial residues (12.5–35.3%)	(1) High bacteria, lowered nutritional quality (2) Adulteration causes lowered nutritional quality and contamination (3) Exposure to unacceptable concentrations of antimicrobial residues	[15]

*N*: sample size in literature from which data were extracted; TBC: total bacterial count; TCC: total coliform count; (): identified risk level – number of positive samples (*n*) or percentage (%).

**Table 2:** Bacterial count (TBC and TCC) and drug residues in milk samples analyzed in this study.

Region	*N*	TBC (±SE × 10^7^ cfu/ml)	TCC (±SE × 10^6^ cfu/ml)	Tetracycline % positive samples (*n*)	Sulphur % positive samples (*n*)
Overall	327	1.0 ± 2.0	1.1 ± 4.6	16.9% (31)	42.1% (77)
Morogoro	103	2.30 ± 2.0^a^	2.6 ± 4.6^a^	25.2% (26)^a^	55.4% (57)^a^
Coast	80	0.06 ± 2.3^b^	0.40 ± 1.5^b^	6.3% (5)^b^	25% (20)^b^
Tanga	144	0.61 ± 2.0^c^	0.22 ± 2.0^c^	ND	ND

*N*: sample size; TBC: total bacterial count; TCC: total coliform count; ND: not detected; (): percentage of samples tested positive on drug residues. Different superscripts within a column indicate statistical significance of values among regions (*P* < 0.05).

**Table 3:** Bacteria detected in milk samples in the present study.

Region	*N*	*EC*	*SS*	*KS*	*PS*	*PA*	*LM*	*LIN*	*LIV*	*SA*	*BA*
Morogoro	103 (87)^1^	6 (6.9)^a^	3 (3.5)	ND	ND	3 (3.5)	47 (54)^a^	20 (22.9)^a^	13 (14.9)^a^	9 (10.3)^a^	11 (12.6)^a^
Coast	80 (50)^1^	ND	ND	ND	ND	ND	21 (42)^b^	8 (16)^b^	2 (4.0)^b^	ND	14 (17.5)^a^
Tanga	144 (101)^1^	12 (11.8)^b^	ND	12 (11.8)	9 (8.9)	ND	ND	13 (21.8)^c^	3 (2.9)^b^	2 (2.0)^b^	32 (31.7)^b^

Total	327 (238)^1^	18 (7.6)	3 (1.3)	12 (5.0)	9 (3.8)	3 (1.3)	68 (28.6)	41 (17.2)	18 (7.6)	11 (4.6)	57 (23.9)

*N*: sample size; *EC: E. coli; SS: Salmonella *spp.*; KS: Klebsiella *spp.*; PS: Proteus *spp.*; PA: P. aeruginosa; LM: L. monocytogenes; LIN: L. innocua; LIV: L. ivanovii; SA: S. aureus; BA: B. abortus; *()^1^: number of positive samples in a region; (): percentage of samples detected with named bacteria in positive samples; ND: not detected. Different superscripts within a column indicate statistical significance of values among regions (*P* < 0.05).

## References

[1] Food and Agriculture Organization (FAO) (2013). *Milk and Dairy Products in Human Nutrition*.

[2] Tschirley D., Reardon T., Dolislager M., Snyder J. (2014). The rise of a middle class in East and Southern Africa: implications for food system transformation. *Working Paper*.

[3] Omamo S. W., Diao X., Wood S. (2006). Strategic priorities for agricultural development in Eastern and Central Africa. *Research Report*.

[4] Ministry of Livestock and Fisheries Development (MLFD) (2011). *Livestock Sector Development Programme*.

[5] MLFD Budget speech and annual estimates of MLFD. http://www.mifugouvuvi.go.tz/budget-speech-20122013/.

[6] Kurwijila L. R., Omore A., Staal S., Mdoe N. S. Y. (2006). Investigation of the risk of exposure to antimicrobial residues present in marketed milk in Tanzania. *Journal of Food Protection*.

[7] International Livestock Research Institute (ILRI) Dairy value chain in Tanzania. Background proposal for CGIAR Research Program on Livestock and Fish. http://cgspace.cgiar.org/bitstream/handle/10568/16962/LivestockFish_DairyVCTanzania.pdf?sequence=1.

[8] Kazwala R. R., Daborn C. J., Kusiluka L. J. M., Jiwa S. F. H., Sharp J. M., Kambarage D. M. (1998). Isolation of Mycobacterium species from raw milk of pastoral cattle of the Southern Highlands of Tanzania. *Tropical Animal Health and Production*.

[9] Mdegela R. H., Kusiluka L. J. M., Kapaga A. M. (2004). Prevalence and determinants of mastitis and milk-borne zoonoses in smallholder dairy farming sector in Kibaha and Morogoro districts in eastern Tanzania. *Journal of Veterinary Medicine Series B: Infectious Diseases and Veterinary Public Health*.

[10] Kivaria F. M., Noordhuizen J. P. T. M., Kapaga A. M. (2006). Evaluation of the hygienic quality and associated public health hazards of raw milk marketed by smallholder dairy producers in the Dar es Salaam region, Tanzania. *Tropical Animal Health and Production*.

[11] Swai E. S., Schoonman L. (2011). Microbial quality and associated health risks of raw milk marketed in the Tanga region of Tanzania. *Asian Pacific Journal of Tropical Biomedicine*.

[12] Kilango K., Makita K., Kurwijila L., Grace D. Boiled milk, food safety and the risk of exposure to milk borne pathogens in informal dairy markets in Tanzania.

[13] Lubote R., Shahada F., Matemu A. (2014). Prevalence of *Salmonella spp*. and *Escherichia coli* in raw milk value chain in Arusha, Tanzania. *American Journal of Research Communication*.

[14] Ngasala J. U. B., Nonga H. E., Mtambo M. M. A. (2015). Assessment of raw milk quality and stakeholders’ awareness on milk-borne health risks in Arusha City and Meru District, Tanzania. *Tropical Animal Health and Production*.

[15] Karimuribo E. D., Gallet P. L., Ng'umbi N. H. (2015). Status and factors affecting milk quality along the milk value chain: a case of Kilosa district, Tanzania. *Livestock Research for Rural Development*.

[16] Tanzania Food and Drugs Authority (TFDA) Government of the United Republic of Tanzania, Tanzania Food, Drugs and Cosmetics Act 2003. http://www.tic.co.tz/media/TFDA%20ACT.pdf.

[17] East African Standard (EAS) Raw Cow Milk Specification in East African Community Standard (EAC 67). https://law.resource.org/pub/eac/ibr/eas.67.2006.html.

[18] Brown H. M. (2014). *Assessment of microbial quality of raw cow’s milk and antimicrobial susceptibility of selected milk-borne bacteria in Kilosa and Mvomero districts, Tanzania [M.S. dissertation]*.

[19] Mdegela R. H., Ryoba R., Karimuribo E. D. (2009). Prevalence of clinical and subclinical mastitis and quality of milk on smallholder dairy farms in Tanzania. *Tydskr South African Veterinary Association*.

[20] Moyo S. J., Maselle S. Y., Matee M. I., Langeland N., Mylvaganam H. (2007). Identification of diarrheagenic Escherichia coli isolated from infants and children in Dar es Salaam, Tanzania. *BMC Infectious Diseases*.

[21] Mdegela R. H., Karimuribo E., Kusiluka L. J. M. (2005). Mastitis in smallholder dairy and pastoral cattle herds in the urban and peri-urban areas of the Dodoma municipality in Central Tanzania. *Livestock Research for Rural Development*.

[22] Shirima G. M., Kazwala R. R., Kambarage D. M. (2003). Prevalence of bovine tuberculosis in cattle in different farming systems in the eastern zone of Tanzania. *Preventive Veterinary Medicine*.

[23] Kifaro G. C., Moshi N. G., Minga U. M. (2009). Effect of sub-clinical mastitis on milk yield and composition of dairy goats in Tanzania. *African Journal of Food, Agriculture, Nutrition and Development*.

[24] Nijsten R., London N., Van den Bogaard A., Stobberingh E. (1996). Antibiotic resistance among Escherichia coli isolated from faecal samples of pig farmers and pigs. *Journal of Antimicrobial Chemotherapy*.

[25] Lee M. H., Lee H. J., Ryu P. D. (2001). Public health risks: chemical and antibiotic residues. *Asian-Australasian Journal of Animal Sciences*.

[26] Wassenaar T. M. (2005). The use of antimicrobial agents in veterinary medicine and implications for human health. *Critical Reviews in Microbiology*.

[27] Walstra P., Geurts T. J., Omen A., Jellema D., Van Boekel M. A. J. S. (1999). *Dairy Technology Principles of Milk Properties and Processes*.

[28] O'Connor A. M., Anderson K. M., Goodell C. K., Sargeant J. M. (2014). Conducting systematic reviews of intervention questions I: writing the review protocol, formulating the question and searching the literature. *Zoonoses and Public Health*.

[29] International Organization for Standardization (ISO) Microbiology of the food chain—horizontal method for the enumeration of microorganisms (ISO 4833-1).

[30] British Standards Institute (BSI) (2007). *BS EN ISO 6785, Milk and Milk Products, Detection of Salmonella spp*.

[31] International Organization for Standardization (ISO) Microbiology of food and animal feeding stuffs—horizontal method for the detection and enumeration of *Listeria monocytogenes*, ISO 11290-1.

[32] International Organization for Standardization (ISO) Microbiology of food and animal feeding stuffs—horizontal method for enumeration of coagulase-positive staphylococci (*Staphylococcus aureus* and other species), ISO 6888-1.

[33] International Organization for Standardization (ISO) Microbiology of food and animal feeding stuffs—horizontal method for the detection and enumeration of *Enterobacteriaceae*, ISO 21528-2.

[34] Herman L., De Ridder H. (1992). Identification of *Brucella spp*. by using the polymerase chain reaction. *Applied and Environmental Microbiology*.

[35] Saiki R. K., Gelfand D. H., Stoffel S. (1988). Primer-directed enzymatic amplification of DNA with a thermostable DNA polymerase. *Science*.

[36] Rijpens N. P., Jannes G., Van Asbroeck M., Rossau R., Herman L. M. F. (1996). Direct detection of Brucella spp. in raw milk by PCR and reverse hybridization with 16S-23S rRNA spacer probes. *Applied and Environmental Microbiology*.

[37] Cleaveland S., Shaw D. J., Mfinanga S. G. (2007). *Mycobacterium bovis* in rural Tanzania: risk factors for infection in human and cattle populations. *Tuberculosis*.

[38] Moyo S., Haldorsen B., Aboud S. (2015). Identification of VIM-2-producing Pseudomonas aeruginosa from Tanzania is associated with sequence types 244 and 640 and the location of blaVIM-2 in a TniC integron. *Antimicrobial Agents and Chemotherapy*.

[39] Seni J., Falgenhauer L., Simeo N. (2016). Multiple ESBL-producing Escherichia coli sequence types carrying quinolone and aminoglycoside resistance genes circulating in companion and domestic farm animals in Mwanza, Tanzania, harbor commonly occurring plasmids. *Frontiers in Microbiology*.

[40] Grace D., Omore A., Randolph T., Kang'ethe E., Nasinyama G. W., Mohammed H. O. (2008). Risk assessment for *Escherichia coli* O157:H7 in marketed unpasteurized milk in selected East African countries. *Journal of Food Protection*.

[41] Ribeiro T. T. B. C., Costa G., da Costa M. (2017). Microbial contamination in industrial tofu. *Ciência Rural Santa Maria*.

[42] Czuprynski C. J., Kathariou S., Poulsen K. P., Gyles C. L., Prescott J. F., Songer J. G., Thoen C. O. (2010). *Listeria*. *Pathogenesis of Bacterial Infections in Animals*.

[43] Guillet C., Join-Lambert O., Le Monnier A. (2010). Human listeriosis caused by *Listeria ivanovii*. *Emerging Infectious Diseases*.

[44] Nádia M., Diane S., Débora O., Mirlei R. E. (2012). Evaluation of microbiological quality of raw milk produced at two properties in the Far West of Santa Catarina, Brasil. *Food and Public Health*.

[45] Momoh A. R. M., Idonije B. O., Nwoke E. O. (2011). Pathogenic bacteria-a probable cause of primary infertility among couples in Ekpoma. *Journal of Microbiology and Biotechnology Research*.

[46] European Food Safety Authority (EFSA) (2009). Technical specifications for the monitoring and reporting of *verotoxigenicEscherichia coli* (VTEC) on animals and food (VTEC surveys on animals and food). *EFSA Journal*.

[47] Karimuribo E. D., Ngowi H. A., Swai E. S., Kambarage D. M. (2016). Prevalence of brucellosis in crossbred and indigenous cattle in Tanzania. *Livestock Research for Rural Development*.

[48] Goossens B., Mbwambo H., Msangi A., Geysen D., Vreysen M. (2006). Trypanosomosis prevalence in cattle on Mafia Island (Tanzania). *Veterinary Parasitology*.

[49] Kurwijila L. R., Mwingira J., Karimuribo E. (2011). *Safety of Animal Source Food in Tanzania, a Report of Situation Analysis for the Safe Food Fair Food Project*.

[50] World Health Organization (WHO) (2008). *The Global Burden of Disease: 2004 Update*.

[51] Joseph E. (2014). *Assessment of microbiological hazards along the milk value chain in Kilosa and Mvomero districts, Tanzania [M.S. dissertation]*.

[52] Shija F. (2013). *Assessment of milk handling practices and bacterial contaminations along the dairy value chain in Lushoto and Handeni districts, Tanzania [M.S. dissertation]*.

[53] Addo K. K., Mensah G. I., Aning K. G. (2011). Microbiological quality and antibiotic residues in informally marketed raw cow milk within the coastal savannah zone of Ghana. *Tropical Medicine and International Health*.

[54] Mhone T. A., Matope G., Saidi P. T. (2012). Detection of *Salmonella* spp., *Candida albicans*, *Aspergillus* spp., and antimicrobial residues in raw and processed cow milk from selected smallholder farms of Zimbabwe. *Veterinary Medicine International*.

[55] Mosu S., Megersa M., Muhie Y., Gebremedin D., Keskes S. (2013). Bacteriological quality of bovine raw milk at selected dairy farms in Debre Zeit town, Ethiopia. *Journal of Food Science and Technology Research*.

[56] Häsler B., Fornace K., Eltolth M., Rushton J. (2014). Rapid assessment of nutrition and food safety risks in dairy value chains in Tanzania. *Project Report*.

[57] Mohammed S., Munissi J. J. E., Nyandoro S. S. (2016). Aflatoxin M1 in raw milk and aflatoxin B1 in feed from household cows in Singida, Tanzania. *Food Additives and Contaminants: Part B Surveillance*.

